# JASPER: A fast genome polishing tool that improves accuracy of genome assemblies

**DOI:** 10.1371/journal.pcbi.1011032

**Published:** 2023-03-31

**Authors:** Alina Guo, Steven L. Salzberg, Aleksey V. Zimin

**Affiliations:** 1 Department of Biomedical Engineering, Johns Hopkins University, Baltimore, Maryland, United States of America; 2 Department of Applied Mathematics and Statistics, Johns Hopkins University, Baltimore, Maryland, United States of America; 3 Center for Computational Biology, Johns Hopkins University, Baltimore, Maryland, United States of America; 4 Department of Computer Science, Johns Hopkins University, Baltimore, Maryland, United States of America; 5 Department of Biostatistics, Johns Hopkins University, Baltimore, Maryland, United States of America; University of Trento, ITALY

## Abstract

Advances in long-read sequencing technologies have dramatically improved the contiguity and completeness of genome assemblies. Using the latest nanopore-based sequencers, we can generate enough data for the assembly of a human genome from a single flow cell. With the long-read data from these sequences, we can now routinely produce de novo genome assemblies in which half or more of a genome is contained in megabase-scale contigs. Assemblies produced from nanopore data alone, though, have relatively high error rates and can benefit from a process called polishing, in which more-accurate reads are used to correct errors in the consensus sequence. In this manuscript, we present a novel tool for genome polishing called JASPER (Jellyfish-based Assembly Sequence Polisher for Error Reduction). In contrast to many other polishing methods, JASPER gains efficiency by avoiding the alignment of reads to the assembly. Instead, JASPER uses a database of k-mer counts that it creates from the reads to detect and correct errors in the consensus. Our experiments demonstrate that JASPER is faster than alignment-based polishers, and both faster and more accurate than other k-mer based polishing methods. We also introduce the idea of using a polishing tool to create population-specific reference genomes, and illustrate this idea using sequence data from multiple individuals from Tokyo, Japan.

## Introduction

As a result of continual increases in sequencing efficiency and much longer read lengths, highly contiguous genome assemblies are now a staple of genomic research. In particular, instruments from Oxford Nanopore Technologies have enabled many scientists to sequence and assemble genomes from many species without the need for a significant investment in sequencing equipment. A single PromethION flowcell, for example, now yields enough data to produce a highly contiguous assembly of a human genome. However, because the per-base accuracy of ONT reads is still relatively low, creating an accurate final sequence (the “consensus”) remains a challenge. One way to improve the consensus quality is to “polish” the assembly using accurate reads from the same DNA source. These more-accurate reads can either be short (100-250bp) Illumina reads, or more expensive but longer (10Kb and above) PacBio HiFi reads. A variety of polishing tools have been developed and published for this purpose, including Pilon [1], Racon [2], POLCA [3], ntEdit [4], and NextPolish [5]. The recently published Merfin tool [6] uses k-mers to filter variant calls that can be later used for polishing. Almost all these tools require aligning the polishing reads to the assembled contigs, which is computationally expensive. ntEdit does not require alignment, but instead uses Bloom filters to compute and store k-mer quality information about the reads used for polishing. K-mer counts and base quality values are also used for correcting errors in Illumina reads in tools such as QUAKE [7] and QuORUM [8].

In this manuscript, we introduce a novel alignment-free polishing tool, JASPER (Jellyfish-based Assembly Sequence Polisher for Error Reduction), which uses k-mer counts computed from the polishing reads with Jellyfish [9] to make corrections in assembled contigs. Our experiments show that JASPER is substantially faster than alignment-based polishing tools, with a relatively small cost in overall accuracy. We also show that JASPER is both faster and more accurate than ntEdit.

The computational efficiency of JASPER enables another possible application, which we illustrate in this study with an example. One can use JASPER to produce population-specific human reference genomes, using Illumina reads sequenced from multiple individuals from a population of interest. Aligning these Illumina reads to a genome could become quite expensive, especially if the number of individuals is large, while counting k-mers in the reads is much cheaper computationally. JASPER can use these k-mer counts to “correct” a human genome assembly so that it contains all homozygous variants that are common in the population from which the reads were drawn. When using this population-specific reference for comparisons of DNA from other individuals from the same population, shared variants will not be seen, thus making downstream analyses easier.

### Design and implementation

JASPER attempts to locate and correct errors in a genome sequence using only the k-mer count data (k = 37 by default) in a set of reads sequenced from the same individual. Given a set of reads, JASPER first calls Jellyfish to count k-mers in the reads. It then uses the “jellyfish histo” command to create a histogram of k-mer counts N(C), where N is the number of distinct k-mers with count C in the reads. The correction algorithm is implemented in Python, and it calls Jellyfish from within Python to load and query the database of k-mer counts. JASPER runs multiple instances of the correction algorithm in parallel, using shared memory to load the database.

**Fig 1:**
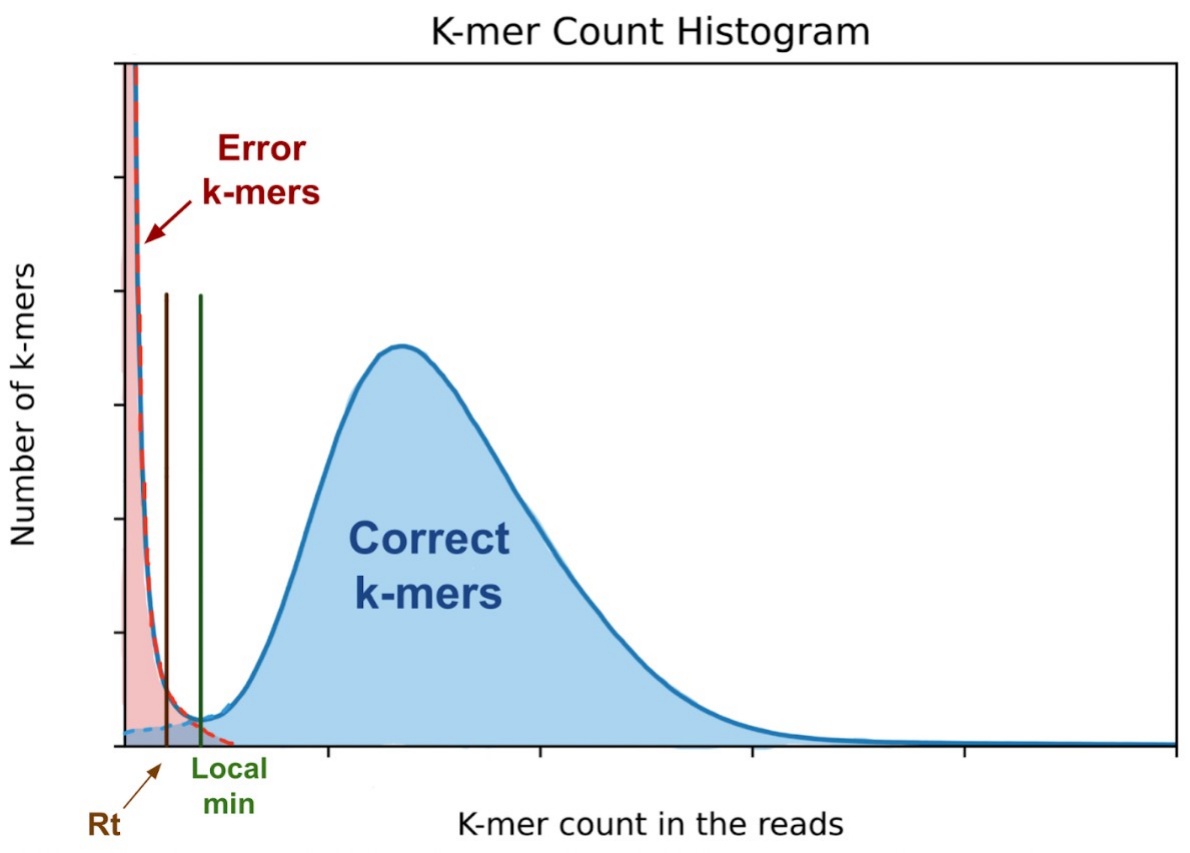
A typical k-mer count histogram for low-error-rate sequencing data (Illumina or PacBio HiFi). The red region contains error k-mers that are due to sequencing errors in the reads. The blue region represents the distribution of the counts of correct k-mers in the reads. The x-position labeled R_t_ is defined as half of the x coordinate of the local minimum of the distribution.

Fig 1 shows a typical k-mer count histogram, with the k-mer count on the horizontal axis and the number of k-mers with a particular count on the vertical axis. The histogram is actually a combination of two different functions E(C) and R(C), where E(C) is the distribution of erroneous k-mers as a function of their count (shown in red in Fig 1) and R(C) is the number of true k-mers (from the genome) that occur in the sequence data (blue area in Fig 1). The local minimum in the histogram occurs at the point C_eq_ where E(C_eq_)~ = R(C_eq_), where any k-mer with a count of C_eq_ has a 50% chance of containing a sequencing error. K-mers with counts below C_eq_ are more likely to contain sequencing errors than to belong to the genome and therefore we call them *unreliable*. Not every unreliable k-mer contains an error, but unreliable k-mers are more likely to contain an error than not.

The general workflow of JASPER is as follows. For each contig sequence in an assembly, we look up the count for each k-mer in the contig in the database of k-mer counts (from the reads). We skip any k-mers containing a non-ACGT character. For each k-mer in the assembly, we determine whether it is unreliable, and thus is likely to contain an error, by using a heuristic approach with two thresholds: an absolute threshold, A_t_, and a relative threshold, R_t_, where A_t_ < R_t_ < C_eq_. Any k-mer with a count below A_t_ is considered to be unreliable. By default, JASPER sets R_t_ = floor(0.5*C_eq_) and A_t_ = round(0.5*R_t_), where C_eq_ is the local minimum of the histogram curve as shown in Fig 1, and floor(x) is the closest integer to x that is smaller than x. We note that this computation works for C_eq_ > = 4. If C_eq_ < 4, then JASPER considers that the input read data is not suitable for polishing and returns an error. If a k-mer has a count less than R_t_ but not less than A_t_, then we label it as unreliable if its count is also less than half of the count of the previous k-mer. These heuristics are intended to account for natural variations of coverage in the sequencing data. As we examine k-mer counts along the genome sequence, any drop in k-mer counts that is due to natural variation in coverage is likely to be smooth, whereas any drop that is due to an error in the sequence is likely to be sharp. When we find the first unreliable k-mer K_e_ after a run of correct k-mers, all subsequent consecutive k-mers whose counts are below R_t_ are considered to be unreliable as well. JASPER continues to mark k-mers as unreliable until it sees a k-mer with a count above R_t_. This generates a “run” of unreliable k-mers of length L. In highly repetitive regions, JASPER limits its efforts by only marking as unreliable a run of K or K-1 consecutive k-mers whose maximum count is 50 times less than the average k-mer counts of the K k-mers before the run. We use the length of the run L to determine if there is a potential error in the contig sequence, the putative error type and the appropriate correction strategy, as follows:

1. L = K (37 by default): the putative error is a single-base substitution or insertion located at the last base of the sequence of errors, S_L_. JASPER attempts to fix this error by changing the last base of S_L_ or deleting it. We accept the fix if all k-mers spanning the putative deletion site have counts of at least R_t_ after the modification.
2. L > K: we assume that this is caused by two or more errors that are less than K apart. In this case we perform the correction by building a k-mer graph. We take the last K-1 bases of the last good k-mer and query the Jellyfish database for the counts of k-mers corresponding to the four possible extensions (A,C,G,T). Any extension that yields a k-mer with count above R_t_ is valid. Each valid extension originates a path that we can extend further. Thus, we build a local directional k-mer graph that contains all paths of k-mers with counts above R_t_. We follow all possible paths for up to L steps. If the number of possible paths exceeds 5,000 at any step, we give up. If the last 5 bases of any path match the first 5 bases of the first good k-mer, we test all k-mers that contain the matching 5 bases, and if all these k-mers’ counts are above R_t_, we accept the path as the fix. By construction this ensures that the counts of all k-mers along the path are above R_t_.
3. L < K: for this scenario the putative error is either (i) a single base deletion somewhere in S_L_; (ii) an insertion of the same base if the last base of the last reliable k-mer before S_L_ is the same as the last base of the first unreliable k-mer (in S_L_); (iii) a haplotype phasing error where the assembly algorithm chose a base from one haplotype and a base from the other haplotype separated by K or fewer positions; or (iv) a deletion of multiple bases that are the same as the last base of the last reliable k-mer before S_L_. We try the modifications sequentially until we arrive at the situation where all k-mers containing bases in modified S_L_ have counts of at least R_t_.

**Fig 2:**
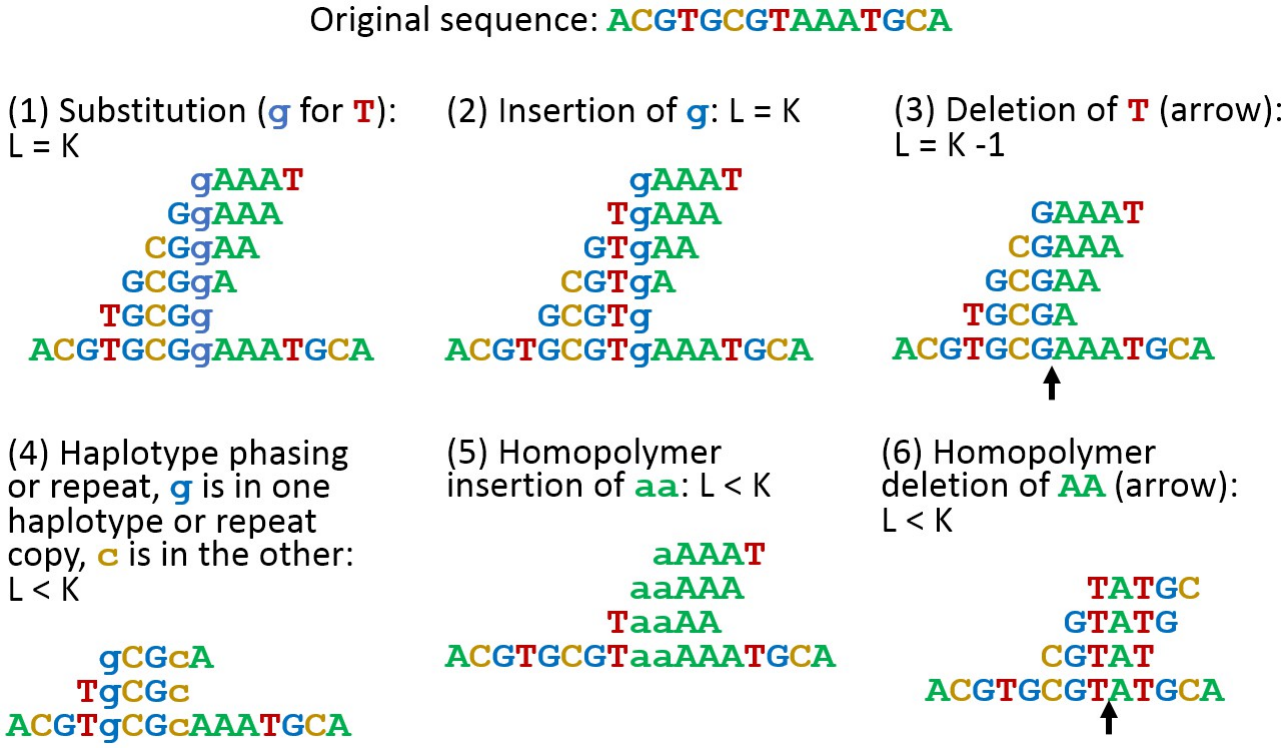
Illustration of the six most common error types that JASPER can detect and fix. Error bases are in lowercase. The error k-mers (K = 5) are shown above the sequence. Black arrows indicate locations of deletions.

The major types of errors described above are illustrated in Fig 2, which uses K = 5 for clarity and space considerations. Note that for haplotype phasing errors, only one of the bases (the last base of the first k-mer in S_L_, or the first base of the last k-mer in S_L_) will be wrong.

If we are unable to find a way to modify the consensus in such a way as to make all k-mers in S_L_ reliable, the algorithm makes no modifications because the fact that a k-mer is unreliable does not guarantee that it is erroneous.

**Fig 3:**
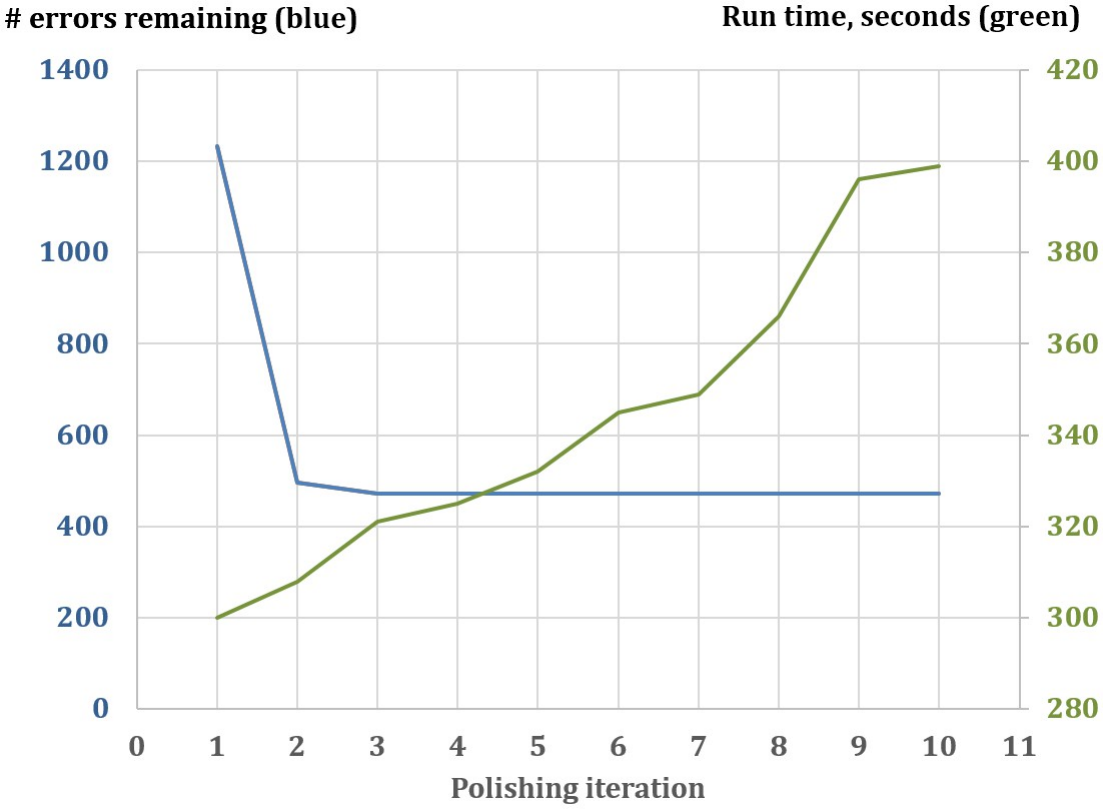
Number of errors remaining after polishing (green, left axis) and the run time of JASPER (green, right axis) for different number of polishing iterations (-p parameter), on *A*. *thaliana* data with simulated errors with K = 63. The number of errors stops decreasing after three iterations, and it never increases for up to 10 iterations. The run time increases approximately linearly with the number of iterations. We do not show the original number of errors (pass 0), because it is large (118,563).

After we make a complete pass of corrections over the entire set of contig sequences, by default we run one more pass. Errors fixed on the first pass may allow JASPER to fix another set of errors on subsequent passes. Note that the number of errors does not increase with more passes, implying that the JASPER algorithm introduces few or no new errors. Fig 3 shows the number of errors remaining in the *A*. *thaliana* simulated data experiment for up to 10 passes of polishing. The number of errors reaches its minimum after three passes, and then flattens out completely, implying that the subsequent passes do not introduce or correct any more errors. We did verify that for this case the number of corrections made in passes 4–10 was indeed zero.

We note the distinction between ntEdit and JASPER algorithms. ntEdit is based on finding a subset of “solid” k-mers in a set of Illumina reads using Bloom filters, and then using this binary designation to detect and fix errors in the consensus. In JASPER we use the actual counts of occurrences of k-mers in the reads used for polishing to determine whether the k-mer is likely to contain an error, depending on the local context, i.e. the difference between its count and the counts of the preceding and following k-mers. Thus, depending on the context, not all unreliable k-mers will be called potentially erroneous and trigger the correction algorithm. Another major difference from ntedit is the use of the k-mer graph method to correct cases where there are several errors close together. These algorithmic differences in JASPER lead to better effectiveness with fewer erroneous corrections, and thus running multiple passes of JASPER leads to uniformly better-polished output assemblies, as shown in Fig 3.

## Results

We first compare JASPER to several leading genome polishing tools, including POLCA [3], NextPolish [5], and ntEdit [4]. POLCA and NextPolish are among the best methods for polishing with Illumina reads [3], and ntEdit is the best of the alignment-free genome polishers.

In our first experiments, we utilized publicly available data for the model plant *Arabidopsis thaliana* (ecotype Col-0, see Data availability section). We began with a previously-published high-quality assembly of the genome [10], which we label Athal-Berlin, and introduced 39635 single base substitutions and 78928 single base insertions and deletions at random locations in the genome. We call the resulting assembly Athal-simerr. We then used wgsim (https://github.com/lh3/wgsim) to simulate 30x coverage by 2x150 paired-end Illumina reads with a 1% error rate. We call these reads the “simulated polishing reads.” We then used the simulated polishing reads to correct the Athal-simerr assembly with POLCA, NextPolish, ntEdit and JASPER. In the second experiment, we used the MaSuRCA assembler [11] (v4.0.6) to create another assembly of *A*. *thaliana* using the PacBio and Illumina reads from Berlin et al. [10]. We call this assembly Athal-MaSuRCA. We polished this assembly with POLCA, NextPolish, ntEdit, and JASPER, using a 60x coverage subset of the Illumina reads from Berlin et al. [10] with all methods. Because k-mer length is a key parameter for JASPER and ntEdit, we ran polishing with k ranging from 27–67 for these two programs and chose the value of k that yielded the best result (the least bases in errors remaining) for each program. We used the default settings for the other polishing parameters. For evaluations, we followed the same approach as was used in [5]. We aligned the Illumina reads used for polishing to the polished assemblies with bwa-mem [12] and called variants with the freebayes software [13]. Any homozygous variant, i.e. any variant where no reads agreed with the assembly, and where two or more reads disagreed, was counted as an error.

Our evaluation of the polishing tools on the *A*. *thaliana* data is shown in Tables 1 and 2. Table 1 shows that JASPER overall corrects more errors than ntEdit but does not correct as many as the alignment-based polishing tools, such as POLCA and NextPolish. On the real data ntEdit increases the number of substitution errors slightly, while both methods reduce the number of insertion/deletion (indel) errors. The primary reason for the inferior performance of the k-mer-based methods is that most errors in Athal-MaSuRCA are indels of several bases in a row. While alignment-based methods are able to correct such errors, k-mer-based methods fail when the indel size is close to the size of the k-mer. Table 2 shows a more in-depth analysis of the specific corrections made by different polishing methods on the simulated data. Alignment-based methods perform well in this analysis, where POLCA is more accurate and NextPolish more sensitive. JASPER has the best precision, followed closely by POLCA. JASPER is superior to ntEdit in both sensitivity and precision in this experiment.

**Table 1:** Comparison of polishing tools on assemblies of *A*. *thaliana*. Numbers correspond to the number of bases in substitutions, insertions, or deletions detected by aligning the reads to the assemblies and calling variants with the freebayes software. Any variant with reference allele frequency 0 and alternative allele frequency >1 was considered an error. For substitution errors we counted the number of bases in substitutions, and for indel errors we counted the number of inserted or deleted bases. Columns labeled “Simulated” refer to experiments where we introduced random errors into a reference, and that used short reads simulated from the unaltered reference for polishing. Columns labeled “Real” refer to experiments that used real Illumina reads to polish a draft assembly of the data. Timing data used a 24-core Intel Xeon Gold 6248R @ 3.0Ghz server with 24 threads. Execution time is given for the real data. The best value in each column (lower is better) is shown in bold.

	Simulated errors #bases in substitutions remaining	Simulated errors #bases in indels remaining	Real errors #bases in substitutions remaining	Real errors #bases in indels remaining	Execution time (minutes)
No polishing	39,635	78,928	1,117	2,507	n/a
POLCA	**76**	**234**	**273**	**459**	42.6
NextPolish	721	1,077	316	520	144.0
ntEdit (best k)	1,532 (k = 35)	1,824 (k = 35)	1,153 (k = 41)	1,888 (k = 41)	8.4
JASPER (best k)	181 (k = 63)	292 (k = 63)	869 (k = 37)	1,814 (k = 37)	5.3

**Table 2:** Statistical analysis of the corrections made by different polishing methods on an *A*. *thaliana* with simulated errors using simulated reads. True Positives are errors that we introduced and that were corrected; False Positives are spurious corrections that polishers made where there were no errors introduced; and False Negatives are errors in the genome that were not corrected. The best numbers in each column are in bold. JASPER is the most accurate polisher with the lowest number of False Positives.

	True Positives	False Positives	False Negatives	Sensitivity	Precision
POLCA	118,296	93	213	**99.9%**	**99.9%**
NextPolish	**118,343**	1,629	**166**	**99.9%**	98.6%
ntEdit (k = 35)	115,803	584	2,706	97.8%	99.5%
JASPER (k = 63)	118,169	**90**	340	99.7%	**99.9%**

**Fig 4:**
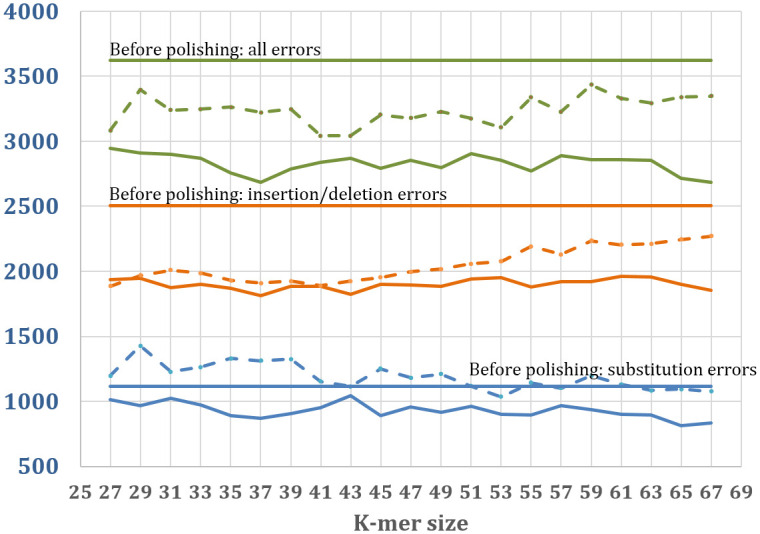
Effectiveness of ntEdit and JASPER polishing on real *A*. *thaliana* data as a function of the k-mer size used for polishing. We show the number of remaining substitution errors in blue, the number of remaining indel errors in orange and the total number of remaining errors in green. Solid horizontal lines mark the number of errors before correction. The number of errors remaining is plotted as solid lines for JASPER and dashed lines for ntEdit. JASPER corrects more substitution errors at every k-mer size, and more indel errors at all but two values of k. For all values of k, the total errors remaining is lower for JASPER.

For a more detailed comparison of JASPER and ntEdit, we varied the k-mer size used for correction from k = 27 to 67 for experiments on real data from *A*. *thaliana*. The results are shown in Fig 4. We observed that with longer k-mers, JASPER’s performance on substitutions improves, while its performance on indels remains flat. For k < = 43, JASPER performs similarly to ntEdit on indels and substantially better on substitutions, resulting in overall better performance at all values of k. Above k = 43, ntEdit’s performance on indels declines (the number of uncorrected errors increases), while its performance on substitutions improves slightly. The run time for JASPER and ntEdit did not vary significantly with increasing k, although the memory usage increased.

*A*. *thaliana* has a relatively small genome that lacks some of the complex repetitive structures that are present in mammalian genomes. To test our approach on a mammalian-sized genome, we used a publicly available but unpublished assembly (GenBank accession GCA_001015355) of the human CHM13 cell line that was assembled with Celera Assembler v. 8.3rc2 from ~70x coverage in PacBio P6C4 reads and polished with Arrow.

We used Illumina reads for CHM13 available from NCBI (accessions SRR1997411 and SRR3189741) for polishing the GCA_001015355 assembly (See Data availability section). The reads provided about 60x coverage of the genome. As with the *A*. *thaliana* real data experiments, we used the approach from [5] to measure the number of errors in the assembly as the number of homozygous variants compared to the Illumina reads after polishing. Table 3 shows polishing results and timings for four programs on the CHM13 assembly. Here JASPER (k = 47) is slower but more accurate than ntEdit (k = 50, recommended value) and both k-mer-based polishers are much faster but less accurate than the alignment-based polishers.

**Table 3:** Comparison of the polishers on the human CHM13 assembly polished with Illumina reads. Timing data used a 24-core Intel Xeon Gold 6248R @ 3.0Ghz server with 24 threads. JASPER and ntEdit are the two fastest polishers. JASPER is slower than ntEdit, but it produces more accurate polished assembly.

	Substitution errors	Insertion/ deletion errors	Execution time (hours)
Unpolished	62,778	298,508	n/a
POLCA	23,118	56,380	13.75
NextPolish	**19,149**	**29,017**	27.5
ntEdit (k = 50)	60,252	220,434	**2.3**
JASPER (k = 47)	59,687	199,583	4.5

JASPER also reports an overall quality value (QV) for the assembly based on the fraction of unreliable k-mers it observes before and after polishing. The Methods section discusses how we determine whether a k-mer is unreliable. We then compute the quality as QV = (-10)log_10_(E_b_), where E_b_ is the proportion of erroneous bases in the assembly. E_b_ is estimated from the observed number of unreliable k-mers as E_b_ = 1—(1—E_k_)^(1/K)^, where K is the k-mer length and E_k_ is the k-mer error rate. E_k_ is computed as the number of unreliable k-mers divided by the genome size; e.g., if we found 100 unreliable k-mers in a 1 Mbp genome, then E_k_ would be 1/10000. This approximation uses a simplifying assumption that the errors are distributed randomly in the genome, which is not generally true; e.g., two or more errors might occur more often consecutively in homopolymers or simple sequence repeats. The QV as computed here is an under-estimate of the true assembly quality, because it assumes that: (1) the reads contain all true k-mers in the genome, and (2) all unreliable k-mers contain an error. Both assumptions are generally not true. In real data sets, it is usually impossible to know how much of the assembled sequence is missing from the reads, especially if the genome was assembled with reads from one sequencing technology (e.g. Oxford nanopore) and polished with reads from another (e.g. Illumina), although that number is likely to be very small when coverage is deep. For example, even in our experiments with 30x coverage of *A*. *thaliana* using simulated 150bp reads, which provide much more uniform coverage than the real data, we found 1080 k-mers (out of about 120M) in the assembly that were not present in the simulated reads.

We can capitalize on the speed of JASPER to address another interesting problem: creating population-specific human reference genomes. When calling short variants (substitutions and indels) from whole-genome sequencing data of one individual against the reference genome, one typically gets several million variant calls. Some of these variant calls, especially those where the individual is homozygous, will be uninformative because they are common variants in the sub-population to which the individual belongs. To reduce the number of uninformative homozygous variant calls, one can use polishing to “correct” the reference genome in places where it disagrees with almost all individuals from a given sub-population. Doing so will essentially hide the variants that are common in the sub-population to which the individual belongs, thus making downstream analysis easier. Polishing tools including Jasper and others can be used create population-specific reference genomes by using Illumina data from multiple individuals to “polish” a human genome assembly such as GRCh38 or CHM13. However, for large population samples the computational cost of making such a reference with alignment-based tools might be prohibitive. In this application, we are not really polishing, but rather altering the reference to make it look much more like an individual genome from the target population. Also, the task here is not to introduce the most common population variants into the reference, but rather remove homozygous variants from the reference that are rare in the population. Due to its efficiency, JASPER is well-suited for this task. To illustrate this capability, we performed two experiments.

In the first experiment, we used the recently published complete CHM13 human genome [14] as the basis for creating a population-specific reference. Because CHM13 is missing the Y chromosome, we added the Y chromosome from GRCh38 to the CHM13 assembly, creating a new assembly which we designate CHM13+Y. We then downloaded Illumina data from eight individuals (NA18939, NA18946, NA18976, NA18978, NA18979, NA18981, NA18991, NA19011), all from Tokyo, Japan, from the 1000 Genomes Project website (http://www.internationalgenome.org). This yielded a total of ~260 Gbp in Illumina reads. We then set aside one sample, NA18939, and used the remaining sets of reads to polish CHM13+Y using JASPER, resulting in an assembly we call Tokyo-polish.

We then aligned the reads from NA18939 to both the CHM13+Y and the Tokyo-polish genomes and called variants with POLCA software utilizing the “-n” switch that switches off all polishing algorithms and simply reports the variant calls in the VCF format [3]. We then counted the number of homozygous variant calls where the reference allele was supported by 0 reads and the alternate allele was supported by at least two reads. We found 1,590,668 such variants when comparing the reads to the CHM13+Y genome, and 1,029,097 variants when comparing to the Tokyo-polish reference. Thus, we observed a 35% reduction in the number of homozygous variant calls against the polished reference.

A related question is whether the polished reference would be closer to an individual who was unrelated to the population of interest. To answer this question, we used the Illumina reads with similar genomic coverage as NA18939 data, for individual PGP17 from the Personal Genomes Project (PGP), who is estimated to be about 60% Ashkenazi with no Japanese ancestry. We observed 1,052,191 homozygous variant calls between the Tokyo-polish reference and the PGP17 individual, vs. 1,029,097 calls between the Tokyo-polish reference and NA18939. Thus (as expected) PGP17 was no closer to the Tokyo-polish assembly than NA18939. We then ran an experiment to compare the use of JASPER to create a population-specific reference versus using the recently-published Ashkenazi Jewish reference genome, Ash1 [15]. Ash1 was assembled from HG002, an Ashkenazi Jewish individual who is part of the 1000 Genomes Project. We also downloaded 30X coverage in Illumina data from his parents, HG003 and HG004. We used the Illumina reads from both parents to “polish” GRCh38.p13, creating a new Ashkenazi-specific reference, which we designated as GRCh38-Ash.

The publication describing Ash1 [15] called variants in the PGP17 genome by aligning the Illumina reads from PGP17 to Ash1, and found 1,333,345 homozygous variants, versus 1,700,364 homozygous variants when found when aligning PGP17 reads to GRCh38. We used the same methods and thresholds to call variants in PGP17 versus our GRCh38-Ash reference genome and found 1,049,117 homozygous variants. Fewer homozygous variants suggest that our polished genome is an even closer match to PGP17 than the individual Ash1 genome for the purpose of short variant calling.

In summary, JASPER is a very fast tool for polishing genomes, which runs many times faster than the fastest alignment-based polisher. In addition to its usefulness as a polishing tool, JASPER can also be used to efficiently create new personalized reference genomes.

### Availability and future directions

In this manuscript we introduced an efficient novel polishing tool called JASPER that is substantially faster than polishing methods based on sequence alignment, and more accurate than currently available k-mer based methods. The efficiency and scalability of JASPER allow one to use it to create personalized reference genomes for specific populations very efficiently, even for large sequenced populations. JASPER is an open-source software available on GitHub at https://github.com/alguoo314/JASPER.

## Supporting information

S1 Text Command lines used for polishing experiments and evaluations. (DOCX)
