# Supplementary material for: JASPER: A fast genome polishing tool that improves accuracy of genome assemblies
Source: PLoS Comput Biol. 2023 Mar 31;19(3):e1011032. doi: 10.1371/journal.pcbi.1011032 (PMC10096238; doi:10.1371/journal.pcbi.1011032)
Supplement: S1 Text — (DOCX) [file pcbi.1011032.s001.docx]

**S1 Text.**

**Command lines used for polishing experiments and evaluations.**  Here are the command lines we used for the *A. thaliana* experiments.

For the simulated data experiments we created 60x coverage by the simulated reads by using wgsim software:

wgsim -N 23999770 -r 0 -1 150 -2 150 -e 0.01 arabidopsis.fa sim_R1.fastq sim_R2.fastq

For ntEdit we used a range of k values between 27 and 67 and ran nthits followed by ntedit. Here is an example command for k=47:

nthits -b 36 -k 47 -t 24 --outbloom -p solidBF --solid sim_read1.fastq sim_read2.fastq

ntedit -f arabidopsis.with_errors.fa -r solidBF_k47.bf -b arabidopsis.ntedit -t 24

For POLCA we used default settings with 24 threads:

polca.sh -a arabidopsis.with_errors.fa -r ‘sim_read1.fastq sim_read2.fastq’ -t 24

For Nextpolish we first aligned the reads to the assembly with the bwa tool and sorted the alignments with samtools followed by running nextpolish:

bwa index arabidopsis.with_errors.fa -p arabidopsis.with_errors.fa.bwa

cat sim_read1.fastq sim_read2.fastq | bwa mem -SP -t 24 arabidopsis.with_errors.fa.bwa 1>alignments.sam

samtools sort -@ 24 <(samtools view -uhS alignments.sam) alignments.alignSorted

samtools faidx arabidopsis.with_errors.fa

samtools index alignments.alignSorted.bam

python nextpolish1.py -g arabidopsis.with_errors.fa -t 1 -p 24 -s alignments.alignSorted.bam > arabidopsis.with_errors.nextpolish1.fa

bwa index arabidopsis.with_errors.nextpolish1.fa -p arabidopsis.with_errors.nextpolish1.fa.bwa

cat sim_read1.fastq sim_read2.fastq | bwa mem -SP -t 24 arabidopsis.with_errors.nextpolish1.fa.bwa 1>alignments.sam

samtools sort -@ 24 <(samtools view -uhS alignments.sam) alignments.alignSorted

samtools faidx arabidopsis.with_errors.nextpolish1.fa

samtools index alignments.alignSorted.bam

python nextpolish1.py -g arabidopsis.with_errors.nextpolish1.fa -t 2 -p 24 -s alignments.alignSorted.bam > arabidopsis.with_errors.nextpolish.fa

For JASPER we used the defaults:

jasper.sh -t 24 -a arabidopsis.with_errors.fa -r ‘sim_read1.fastq sim_read2.fastq’ ,

For human CHM13 experiments we use the following command lines:

JASPER:

/bin/time jasper.sh -k 47 -t 24 -r ‘SRR1997411_1.fastq SRR1997411_2.fastq SRR3189741_1.fastq SRR3189741_2.fastq’ -a GCA_001015355/GCA_001015355.1_CHM13_CA_Sensitive_5_Error_genomic.fna 1>jasper.out 2>&1

ntEdit:

/bin/time nthits -b 36 -k 50 -t 24 --outbloom --solid SRR1997411_1.fastq SRR1997411_2.fastq SRR3189741_1.fastq SRR3189741_2.fastq 1>nthits.out 2>&1

/bin/time ntedit -f GCA_001015355/GCA_001015355.1_CHM13_CA_Sensitive_5_Error_genomic.fna -r solidBF_k50.bf -b ntEditEcolik25 -t 24 1>ntedit.out 2>&1

POLCA:

/bin/time polca.sh -t 24 -r ‘SRR1997411_1.fastq SRR1997411_2.fastq SRR3189741_1.fastq SRR3189741_2.fastq’ -a GCA_001015355/GCA_001015355.1_CHM13_CA_Sensitive_5_Error_genomic.fna 1>polca.out 2>&1

NextPolish:

For the first pass we used the bam alignment file produced during POLCA polishing:

/bin/time python nextpolish1.py -g GCA_001015355/GCA_001015355.1_CHM13_CA_Sensitive_5_Error_genomic.fna -t 1 -p 24 -s GCA_001015355/GCA_001015355.1_CHM13_CA_Sensitive_5_Error_genomic.fna.alignSorted.bam > GCA_001015355/GCA_001015355.1_CHM13_CA_Sensitive_5_Error_genomic.fna.pass1

We then ran POLCA with -n switch to re-align the reads to the output of pass1:

/bin/time polca.sh -t 24 -r ‘ SRR1997411_1.fastq SRR1997411_2.fastq SRR3189741_1.fastq SRR3189741_2.fastq ‘ -a GCA_001015355/GCA_001015355.1_CHM13_CA_Sensitive_5_Error_genomic.fna.pass1 -n 1>polca.out 2>&1

After than we ran the second pass with the resulting bam file:

/bin/time python nextpolish1.py -g GCA_001015355/GCA_001015355.1_CHM13_CA_Sensitive_5_Error_genomic.fna.pass1 -t 1 -p 24 -s GCA_001015355/GCA_001015355.1_CHM13_CA_Sensitive_5_Error_genomic.fna.pass1.alignSorted.bam > GCA_001015355/GCA_001015355.1_CHM13_CA_Sensitive_5_Error_genomic.fna.pass2

All evaluations:

We ran POLCA with -n switch as a convenient single-command way to produce the variant calls in the vcf format. -n switch disables all polishing and simply runs “bwa index”, “bwa mem” , “samtools sort” followed by parallelized variant calling with freebayes software.

/bin/time polca.sh -t 24 -r ‘SRR1997411_1.fastq SRR1997411_2.fastq SRR3189741_1.fastq SRR3189741_2.fastq’ -a polished_assembly.fa -n 1>/dev/null 2>&1

We then used the following awk script to count bases in homozygous substitutions/indels:

awk 'BEGIN{ind=0;subs=0;}{split($10,a,":");ro=a[4];ao=a[6];if(ao>=2 && ro==0){if(length($4)==length($5)){subs+=length($4)}else{t=length($4)-length($5);if(t<0){t=-t}ind+=t}}}END{print "Subs:"subs" Ind:"ind}' polished_assembly.fa.vcf > polished_assembly.fa .report.txt

For the Tokyo human cohort experiments we ran jasper one the following files downloaded from NCBI SRA:

jasper.sh -t 24 -a CHM13.withY.fa -r 'ERR052929_2.fastq ERR052929_1.fastq SRR768164_1.fastq SRR768164_2.fastq SRR768165_1.fastq SRR768165_2.fastq SRR359110_1.fastq SRR359110_2.fastq ERR251634_1.fastq ERR251633_1.fastq ERR251633_2.fastq ERR251634_2.fastq SRR741404_2.fastq SRR741403_2.fastq SRR741404_1.fastq SRR741403_1.fastq SRR359083_1.fastq SRR359083_2.fastq ERR234334_1.fastq ERR234334_2.fastq'

We counted the number of homozygous variants in the human cohort experiments by aligning the reads from sample NA18939 reads 'SRR768162_2.fastq SRR768163_1.fastq SRR768163_2.fastq SRR768162_1.fastq' to T2T CHM13 assembly with chrY from HG002 CHM13.withY.fa before and after polishing with JASPER using the “-n” option in POLCA polisher, which conveniently runs the alignment of reads and variant calling by freebayes in a single command. For PGP17 genome we used read files (hu34D5B9/fastq/56001801066384A_1.fq and hu34D5B9/fastq/56001801066384A_2.fq). We then counted the number of homozygous variants with allele frequency >=2 using the following command:

grep -v '^#' output.vcf |awk '{split($10,a,":");if(a[4]==0 && a[6]>=2) print $0}' |wc -l

For Ashkenazi experiments we used GRCH38.noalts.fa assembly (GRCh38 without alternative scaffolds). We “polished” the assembly with jasper with default settings using Illumina reads from HG003 and HG004. We then again used POLCA with -n flag to call variants in PGP17 data (hu34D5B9/fastq/56001801066384A_1.fq and hu34D5B9/fastq/56001801066384A_2.fq) against Ash1 genome, and against polished GRCh38.noalts.fa.
